# Distinction between hand dominance and hand preference in primates: a behavioral investigation of manual dexterity in nonhuman primates (macaques) and human subjects

**DOI:** 10.1002/brb3.160

**Published:** 2013-08-02

**Authors:** Pauline Chatagny, Simon Badoud, Mélanie Kaeser, Anne-Dominique Gindrat, Julie Savidan, Michela Fregosi, Véronique Moret, Christine Roulin, Eric Schmidlin, Eric M Rouiller

**Affiliations:** Unit of Physiology Department of Medicine Faculty of Sciences and Fribourg Center for Cognition, University of FribourgChemin du Musée 5, CH-1700, Fribourg, Switzerland

**Keywords:** Bimanual coordination, handedness, intermanual difference, motor performance, precision grip

## Abstract

**Background** The present study aimed to determine and confront hand preference (hand chosen in priority to perform a manual dexterity task) and hand dominance (hand with best motor performance) in eight macaques (*Macaca fascicularis*) and in 20 human subjects (10 left-handers and 10 right-handers). **Methods** Four manual dexterity tests have been executed by the monkeys, over several weeks during learning and stable performance phases (in controlled body position): the modified Brinkman board, the reach and grasp drawer, the tube and the bimanual board tasks. Three behavioral tests, adapted versions from the monkeys tasks (modified Brinkman board, tube and bimanual board tasks), as well as a handedness questionnaire, have been conducted in human subjects. **Results** In monkeys, there was a large disparity across individuals and motor tasks. For hand dominance, two monkeys were rather right lateralized, three monkeys rather left lateralized, whereas in three monkeys, the different parameters measured were not consistent. For hand preference, none of the eight monkeys exhibited a homogeneous lateralization across the four motor tasks. *Macaca fascicularis* do not exhibit a clear hand preference. Furthermore, hand preference often changed with task repetition, both during training and plateau phases. For human subjects, the hand preference mostly followed the self-assessment of lateralization by the subjects and the questionnaire (in the latter, right-handers were more lateralized than left-handers), except a few discrepancies based on the tube task. There was no hand dominance in seven right-handers (the other three performed better with the right hand) and in four left-handers. Five left-handers showed left-hand dominance, whereas surprisingly, one left-hander performed better with the right hand. In the modified Brinkman board task, females performed better than males, right-handers better than left-handers. **Conclusions** The present study argues for a distinction between hand preference and hand dominance, especially in macaque monkeys.

## Introduction

How is handedness defined? Commonly, handedness means hand preference. For most people, the preferred hand is the hand which is most efficient to perform specific manual dexterity tasks (e.g., writing, manipulating objects or tools, etc.). In the present study, in line with a previously proposed concept (e.g., Hopkins et al. 1992; Triggs et al. 2000), we propose to emphasize the distinction between two hand attributes: hand preference and hand dominance.

The hand of preference is defined as the hand with which subjects prefer to work on a specific task, instinctively and without concern whether this hand is actually the most efficient one. In bimanual tasks for instance (e.g., tapping a nail with a hammer, knitting, eating with a fork, and a knife, etc.), the preferred hand is the hand which executes the most complex action or the manipulative role, whereas the nonpreferred hand acts mainly as postural support. In the above mentioned bimanual tasks, they need to be learned, whereas other bimanual tasks are more instinctive and they are also observed in nonhuman primates (e.g., peeling a fruit, cracking a nut with a stone, etc.). In contrast to hand preference, hand dominance refers to the hand which shows the best efficiency to perform a particular unimanual action (Serrien et al. 2006), thus reflecting an intermanual difference of motor performance. The general aim of the present study was to assess separately hand preference and hand dominance in eight adult long-tailed macaque monkeys (*Macaca fascicularis*) and in 20 young adult human subjects.

Population-level right-handedness (preference for the right hand) was considered for a long time as a feature of human being (Raymond et al. 1996). During the last 20 years, several studies demonstrated that handedness for specific manual tasks is also present in nonhuman primates, from prosimians to great apes (e.g., Masataka 1989; Ward et al. 1990, 1993; Fagot and Vauclair 1991; Spinozzi et al. 1998; Lacreuse et al. 1999; Hopkins et al. 2011). Whereas 90% of humans are right-handed (Coren and Porac 1977; Raymond and Pontier 2004), the percentage and the direction of the lateralization vary among the nonhuman primates (see e.g., Papademetriou et al. 2005; mainly for reaching tasks). Concerning the great apes, a recent study by Hopkins et al. (2011) showed population right-handedness, except for Orangutans, which tend to use preferentially the left hand. These results are consistent with other studies (Lacreuse et al. 1999; Wesley et al. 2001; Hopkins et al. 2002, 2003, 2004, 2005; Sherwood et al. 2007). Baboons were also found to be right-handed at population level (Fagot and Vauclair 1988; Vauclair et al. 2005). However, some divergent observations were reported (Pouydebat et al. 2010), concluding to the difficulty to establish a stable handedness among Gorillas, based on different behavioral tasks. In Old World monkeys, handedness seems to be less consistent among the family (Westergaard et al. 1997, 2001a,b), as it appears to depend on the species, especially in Macaques. Although some macaques, such as *Macaca mulatta*, exhibited population-level left-handedness when they performed a specific task (also *Macaca fuscata*, see Murata et al. 2008), other species like *M. fascicularis* did not exhibit any manual bias at the population-level for the same tasks (tube task, reaching to food morsel; Westergaard et al. 1997, 2001a,b; see also Lehman 1980b). The above data for *M. mulatta* are not consistent with previous observations derived from food reaching tests (Lehman 1978a), which showed roughly equal numbers of right- and left-handed individuals. Furthermore, the latter author and others reported that handedness was accentuated with monkeys' age, as well as with task repetition (e.g., Lehman 1978a,b, 1980a,b; Westergaard and Suomi 1996; Westergaard and Lussier 1999; Zhao et al. 2012). Similarly, Hopkins (2004) found a less prominent handedness among Old and New World monkeys in comparison to the great apes. It is, however, interesting to highlight that, for some investigators (e.g., Lehman 1980a, 1989; Hopkins et al. 1989; Fagot and Vauclair 1991; Uomini 2009), these disparate results may depend on the task used to determine handedness (see also Spinozzi et al. 1998, 2007). Indeed, these authors showed that the complexity of the task plays an important role. A high-level manual activity involves, most of the time, a manual bias at the population-level, whereas a simple and low-level task does not. A typical example of high-level manual performance is the precision grip (opposition of thumb and usually index finger to grasp an object), requiring the cooperation of several muscles of hand and arm, tendons, ligaments, and the stabilization of the upper limb to ensure a better effectiveness (e.g., Lemon 1993, 2008; Porter and Lemon 1993). Bimanual tasks are considered as high-level ones, involving a coordination of different limbs and movements. As demonstrated in squirrel monkeys, hand preference is correlated to an asymmetry in functional topography of motor cortex between the two hemispheres, with a greater distal forelimb representation in the dominant hemisphere, opposite the preferred hand (Nudo et al. 1992). Asymmetries in the primary motor cortex related to handedness was reported in great apes (Hopkins and Pilcher 2001; Hopkins et al. 2002, 2010; Hopkins and Cantalupo 2004; Dadda et al. 2006; Sherwood et al. 2007) and in humans (e.g., Dassonville et al. 1997).

Hand preference and hand dominance were each determined based on three adapted manual tasks, which belong to high-level manual activities, for both human subjects and monkeys (*M. fascicularis*). Two tests are bimanual coordinated tasks: the bimanual Brinkman board task (Mark and Sperry 1968) and the tube task (Hopkins 1995), whereas the third test is the modified Brinkman board task (original test: Brinkman and Kuypers 1973; see also Brinkman 1984), performed either unimanually or with both hands at the same time. Monkeys had to perform an additional task, the reach and grasp drawer task, whereas humans had to answer a handedness questionnaire, which allowed us to confirm the self-assessment of each subject and, then, to compare the self-assessment with the results derived from the manual dexterity tests. More specifically, the aim of the study was to test the hypothesis that, in *M. fascicularis*, hand preference is variable across tasks and individuals, the dominant hand does not systematically correspond to the preferred hand, whereas human subjects exhibit more systematic lateralization (hand preference) and the preferred hand generally corresponds to the most dexterous hand (dominant hand).

## Material and Methods

### Nonhuman primate subjects

The experiments were conducted on eight adult female monkeys (*M. fascicularis*), aged between 6 and 7 years old at the beginning of the tests (weight: 3–3.9 kg) and housed in 45 m^3^ rooms with four other animals. The monkeys were neither food nor water deprived (see e.g., Kaeser et al. 2010; Schmidlin et al. 2011). None of the animals had executed the different manual dexterity tasks before, so they were totally naïve. The experimental protocol has been approved by the local ethical committee on animal experimentation and it was in accordance with the *Guidelines for the Care and Use of Laboratory Animals* (ISBN 0-309-05377-3; 1996), as well as authorized by local (Canton of Fribourg) and federal (Swiss) veterinary authorities. The present experiments were covered by the official authorization numbers FR 192/07E, FR 206/08, FR 17/09, FR 18/10, FR 22010. The experimental procedures were designed to minimize pain and suffering for the animals. In the part of the present study on monkeys, the protocol was restricted to behavioral assessment, without any surgical or pharmacological intervention. The macaque monkeys originate initially from an officially recognized breeding center in China and were imported via a quarantine center in Europe (Harlan, Milano, Italy), where they stayed during a few months within a large group of a couple of dozen animals from the same origin. After arrival in our animal facility, the animals were habituated during 1–2 months to the new environment, before starting the habituation procedure (2–3 months duration) aimed at transferring the monkey on a free-will basis to the primate chair (see Schmidlin et al. 2011). The present behavioral experiments were then initiated when the monkeys were comfortable with the primate chair.

During each behavioral test, the monkey sat in a primate chair (see Schmidlin et al. 2011), made of Plexiglas® (Transparent PVC, Notz Plastik AG, Biel, Switzerland), with an adjustable opening on top allowing free head movements although the monkey is restrained. The primate chair also comprises two independent sliding doors at the front, allowing execution of manual dexterity tasks with both hands, separately or simultaneously (Schmidlin et al. 2011). Each experimental session was recorded with one to three digital video cameras, depending on the task (drawer, tube, and bimanual board tasks with one camera; modified Brinkman board task with three cameras; Schmidlin et al. 2011). The duration of a typical daily behavioral session was about 60 min and the experiments were conducted with background music to cover possible disturbing, external noise. At the end of the session, the animals received their daily ration of food, composed of cereals, fruits, and vegetables, in addition to the rewards (food pellets) received during the tests.

### Human subjects

The human subjects were 20 persons (students) aged between 18 and 30 years old. The human experiments were conducted in the context of practical courses for students at the University of Fribourg and the subjects gave their full consent to the experimental protocol. They agreed that the data may be used anonymously for the present study. The human subjects first declared themselves either as left- or as right-handers and it corresponded to the hand they used to write. Based on this initial self-declaration, there were ten left-handers (six men and four women) and ten right-handers (four men and six women). The size of each of these two groups (*n* = 10) was chosen as to approximately match the group size of monkeys (*n* = 8). Given the human population bias for right-hand preference (about 90%), self-declared left-handers were deliberately recruited, thanks to a large pool of students available on the campus. It is expected that the self-declared left-handers are less lateralized than the self-declared right-handers.

Each human subject was enrolled in a single behavioral session (lasting about 60–90 min) and he/she executed three manual dexterity tasks, before responding to the handedness questionnaire at the end of the session. The set-ups for the three manual dexterity tasks were positioned on a table and the behavioral session was recorded with a digital video camera. The subjects began with the modified Brinkman board task, followed by the bimanual board task, and finally, the tube task. Before the beginning of the tests, the subjects sat on a chair in the middle and in front of the experimental table. They had to adjust the height of the chair to feel comfortable.

### Behavioral tasks

The assessment of handedness was based on a palette of behavioral manual dexterity tasks, in which macaque monkeys (*n* = 8) and human subjects (*n* = 20) were enrolled. For both monkeys and human subjects, typical video sequences illustrating the various behavioral tasks described below can be visualized on the following website: http://www.unifr.ch/neuro/rouiller/research/PM/pm1.html.

#### Modified Brinkman board task

**Figure 1:**
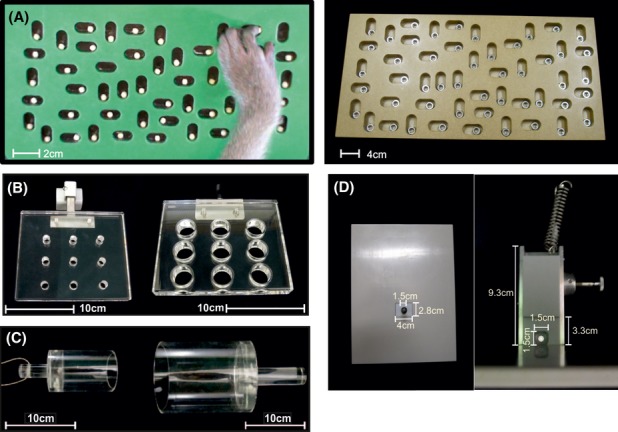
Pictures illustrate the experimental set-ups used in the different behavioral tasks for monkeys and for human subjects. In panel (A), the modified Brinkman board used for monkeys is shown on the left, with each slots filled with a banana pellet, whereas its version adapted for human subjects is shown on the right with each slot filled with a bolt. See text for dimensions of the board and slots. Panel (B) shows the bimanual Brinkman board used for monkeys (on the left) and for humans (on the right). Similarly, in panel (C), the tube used for monkeys is shown on the left and the version adapted for humans on the right. See text for dimensions of the boards, slots, and tubes. In panel (D), the bimanual reach and grasp drawer set-up (used for monkeys only) is shown in a front view (left picture) and from top (right picture). In the top view, the slot in the drawer is clearly visible (with one white pellet inside), as well as the spring at the back of the drawer, imposing to hold the drawer open with one hand while grasping the pellet with the other hand.

The modified Brinkman board and its different adapted versions from the original test of Brinkman and Kuypers (1973) were used routinely for behavioral and motor control studies in macaques (Brinkman 1984; Rouiller et al. 1998; Liu and Rouiller 1999; Freund et al. 2009; Kaeser et al. 2010, 2011, 2013; Schmidlin et al. 2011). The modified Brinkman board for monkeys (Fig. 1A, left panel) is made of a rectangular board of Perspex® with 50 rounded rectangular slots: 25 slots are oriented horizontally and 25 vertically. Each slot measures 6 mm deep, 14 mm long, and 7 mm wide. The board itself measures 22 cm length, 12 cm wide, and 1.2 cm thick. At the beginning of the test, each slot is filled with a banana or sugar flavored pellet (diameter 4 mm). The size of slots permits the monkeys to grasp the pellets only by performing the precision grip, generally using the thumb and the index finger (or rarely another finger, with a flexion of the distal phalanx). Retrieval from the horizontal slots is more difficult than from the vertical ones, because it involves also a rotation of the wrist, either a radial deviation or an ulnar deviation, depending on the position of the corresponding slot on the board (Freund et al. 2009). The board was positioned in front of the monkey with 40° of inclination from horizontal. During each daily session, the animal has used firstly both hands, then each individual hand successively by alternating daily the hand used first. The daily protocol for this task thus comprises three consecutive tests, with retrieval of 50 pellets in each, lasting overall about 10 min, including the time interval to refill the modified Brinkman board with pellets in between the three tests. With respect to the board, the monkey was placed in a middle position (when performing the task with both hand simultaneously), or slightly at the left, or at the right, when using only the right or the left hand, respectively, in such a manner that the hand performing the task is aligned to the set-up. Video sequences illustrating this task can be visualized on the website: http://www.unifr.ch/neuro/rouiller/research/PM/pm1.html (video sequences 1–3) or in a recent visualized experimental report (Schmidlin et al. 2011).

The Brinkman board model, adapted for human subjects (Fig. 1A, right panel), is made of a wooden board of 58 cm long and 28.5 cm wide and it comprises 50 rounded rectangular slots of 4.3 cm long, 2.2 cm wide, and 1.8 cm deep (25 oriented vertically and 25 oriented horizontally). It is tilted with a 30-degree angle from horizontal. Before the beginning of a session, each slot is filled with a bolt (external diameter: 1.8 cm, internal diameter: 1 cm). The bolts replace the food pellets used for the same tests on monkeys. The slots were designed in a manner that subjects have to use the precision grip to retrieve the bolts, and their spatial arrangement is identical to that of the modified Brinkman board used for monkeys. In a single behavioral session, the human subjects had to execute the grasping of the 50 bolts as fast as possible, taking one bolt at a time, and putting it into a plastic box located in front of the board in a middle position. The human subjects were not allowed to throw the bolt into the box. These rules contributed normalizing the test. The subjects performed the task 20 times, using alternatively 10 times the right hand and 10 times the left hand (right, left, right, etc.). The experimenter determined with which hand the subject had to begin (see http://www.unifr.ch/neuro/rouiller/research/PM/pm1.html [video sequences 4–5]).

#### Bimanual Brinkman board task

This task was adapted from the bimanual coordinated task of Mark and Sperry (1968). Our bimanual board is made of transparent acrylic glass (PMMA or Plexiglas®); Fig. 1B). The model for monkeys (Fig. 1B, left panel) measures 15.8 cm long, 13.1 cm large, and has a thickness of 2 cm. It comprises nine holes. Each hole has an upper diameter of 9.5 mm and a lower diameter of 7 mm and contains a sticky reward, like sultana or a little piece of apple. The board is fixed with an inclination of 20–30° from horizontal. The primate chair was placed in the front of the board and the two sliding doors were opened to allow access with both hands simultaneously. The monkeys had to retrieve the reward using both hands at the same time and following one or the other of two possible strategies (see below: analysis of data). One daily session included three to five repetitions of the whole board, with retrieval of each reward. Each hole represented an individual trial (see http://www.unifr.ch/neuro/rouiller/research/PM/pm1.html [video sequence 6]).

The model of the bimanual board adapted for human subjects (Fig. 1B, right panel) is a transparent acrylic glass board of 16 cm long, 13 cm wide, 2 cm thick, and comprising nine holes (diameter of 2.2 cm). The board is fixed with 30° of inclination from horizontal. Before the test started, each hole was filled with a pellet in modeling clay. Using both hands, the human subjects had to take only one pellet at a time and to put it into a plastic box placed in the front of the board. In one session, the subject had to empty the board 20 times. Each hole represented an individual trial (see http://www.unifr.ch/neuro/rouiller/research/PM/pm1.html [video sequence 7]).

#### The tube task

This bimanual task was inspired by the tube task of Hopkins (1995), used to determine handedness in Chimpanzees and later in Old World monkeys (Zhao et al. 2012). Our tube, in transparent acrylic glass (PPMA or Plexiglas®), was adapted to macaques with the following dimensions: the handle measures 4 cm long and 2 cm diameter, the tube itself is 9 cm long from the outside and 7 cm deep from the inside, with an external diameter of 6 cm and an internal diameter of 5 cm. At the bottom of the tube, there is a slot of 0.5 cm in diameter and 0.7 cm deep (Fig. 1C; left panel). The slot was filled with a sticky reward like sultana or little pieces of apple. The tube was attached to a rope by the handle and hung, in such a way that it was placed in front of the primate chair, aligned with the central bar between the sliding doors. The basis of the tube was positioned at the level as the basis of the sliding doors. The test was performed with the two sliding doors open and the animal had to hold the suspended tube with one hand while reaching the reward in the tube with the other hand and bring it to the mouth. A daily session comprised 10–20 trials (see http://www.unifr.ch/neuro/rouiller/research/PM/pm1.html [video sequence 8]).

The model of the tube adapted for human subjects is also made of acrylic glass tube (PPMA or Plexiglas®) with the following dimensions (Fig. 1C, right panel): the tube itself measures 14.7 cm long, 12.8 cm deep, with an external diameter of 12 cm and an internal diameter of 11 cm. The handle is 9.5 cm long and has a diameter of 3 cm. The slot positioned at the bottom of the tube is 2.2 cm in diameter and 0.9 cm deep. The reward was a candy (*Yupi strawberry kiss* or *Yupi MarshMallow*). A second tube was available for human subjects with smaller hands: the dimensions are the same, except the external diameter of 9 cm and the internal diameter of 8 cm. The tube was positioned vertically on the table, with the handle upwards. Starting with the hands placed on the table on each side of the tube, the human subjects had to collect the reward from the tube using both hands. They had the possibility to eat the reward or to give it to the experimenter. Then, the human subjects had to put the tube back on the table at its initial location. The task was performed 20 times to complete the session. One trial was achieved when the human subjects grabbed the tube with one hand while, simultaneously, they took the reward with the other hand (see http://www.unifr.ch/neuro/rouiller/research/PM/pm1.html [video sequence 9]).

#### Reach and grasp drawer task

This bimanual task was used for the monkeys only and it is a simplified version of the set-up previously described (Kazennikov et al. 1994; Kermadi et al. 1998, 2000; Schmidlin et al. 2011). The primate chair was placed in front of the drawer with both sliding doors opened, so that the monkey used both hands. Because of a spring mechanism, once open, the drawer had to be maintained with one hand to avoid that it closed back, while the monkey used the other hand to grasp the pellet, which was initially placed in a slot dig inside the drawer. The dimensions of the object are indicated on the Figure 1D. During one session, the animal executed about five to 15 trials. One trial was achieved when the monkey opened the drawer with one hand, kept it open, and grasped the pellet with the other hand (see http://www.unifr.ch/neuro/rouiller/research/PM/pm1.html [video sequence 10]).

### Handedness questionnaire

At the end of the manual dexterity tasks, the human subjects were asked to answer a handedness questionnaire, elaborated by MacManus (2009). It was chosen because it fills several pertinent criteria to assess handedness in human subjects (Oldfield 1971). The questions dealt with actions of daily life such as: with which hand do you write, do you hold a potato while you are peeling it, do you throw a ball, etc.

### Analysis of data

The data of the behavioral tasks were analyzed manually from the recorded video sequences. The software VirtualDubMpeg2® (Developper Avery Lee, free software, http://www.virtualdub.org) allowed visualizing the video sequences frame by frame, corresponding to a time resolution of 40 msec (acquisition at 25 frames per second). The data were processed first in Excel® worksheets, before they were transferred to Sigmastat®/Sigmaplot® (Systat Software Inc., http://www.sigmaplot.com) and SPSS® (SPSS Inc., Chicago, IL) allowing more elaborated graphic representation and statistical analysis.

The hand dominance was determined based on a single task, the modified Brinkman board task performed with one hand imposed at a time. Two types of data were analyzed for the monkeys (Schmidlin et al. 2011). (i) The score, defined as the number of pellets correctly retrieved during the first 30 sec; (ii) The contact time (CT), defined as the time interval between the first contact of a finger (most often the index finger) with the pellet and the moment when the fingers left the slot with the reward. The CT is a pertinent parameter in addition to the score, as the latter can sometimes be biased. Indeed, the animal may be disturbed by external noises, or may exhibit a lack of motivation or concentration. In such cases, the monkey may interrupt the test, leading to a distortion of the score. Moreover, the CT truly measures the actual manipulation of the pellets with the fingers. The CT was measured for the first five horizontal and the first five vertical slots in the 20 last daily sessions at plateau, whereas the score was calculated for every daily session. The onset of the plateau was defined, when the learning curve tended to saturate (as estimated by visual inspection), as the first value in the nearly flat curve of the score that was not exceeded by one of the five following score values. For human subjects, the analysis of hand dominance was based mainly on the score in 30 sec, although the CT was also established for comparison in a sample of subjects.

The hand preference for monkeys was determined based on four tests: the modified Brinkman board task, when the animal was free to use both hands simultaneously, the reach and grasp drawer task, the tube task, and the bimanual Brinkman board task. For human subjects, two tests were considered, the tube task and the bimanual Brinkman board task, as well as the questionnaire indicating their self-assessed hand preference. For the tube task, the preferred hand was defined as the hand used to grasp the reward into the tube, playing the manipulative role, whereas the other hand, holding the tube, played the postural role. The preferred hand (left hand or right hand) was determined for each tube task trial performed by the subject (humans and monkeys), in order to calculate the handedness index (HI) (see below). For the bimanual board task, the subjects (humans and monkeys) used two different strategies to retrieve the reward. In the first one, the hand above the board pushed the reward while the other hand collected it below the board. In the second one, the hand positioned below the board pushed up the reward using one finger (usually the index finger) and the other hand grasped it above the board, performing the precision grip. In the first strategy (adopted in more than 98% of trials in five out of eight monkeys), the preferred hand is the one pushing the reward. Indeed its role is manipulative, whereas the role of the other hand is postural. For the second strategy, the preferred hand is the one retrieving the reward, as its action is more manipulative and more challenging (precision grip), as compared to the role of the other hand (one finger used). Additionally, the board has an inclination, making this movement still more difficult. This second strategy was used in about half of the trials in one monkey (Mk-MI) and it was predominant in two other monkeys (Mk-CA and Mk-AN; 68% and 98%, respectively). For the reach and grasp drawer task (in monkeys only), the preferred hand is the hand grasping the reward (manipulative role) while the other hand, the postural one, holds the drawer.

For these three tasks (bimanual Brinkman board task, reach and grasp drawer task, tube task), we computed the HI (Westergaard et al. 1997; Spinozzi et al. 1998; Hopkins et al. 2004; Schmitt et al. 2008), defined as follows: the number of trials the right hand (R) was used as preferred hand minus the number of times the left hand (L) was used as preferred hand, divided by the total number of trials:


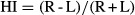


Consequently, a negative HI reflects a left bias whereas a positive HI reflects a right bias. The HI (lateralization) ranges between +1 (strongly right-handed) and −1 (strongly left-handed).

For the modified Brinkman board task, we measured the score in 30 sec when the animal was free to use both hands, and counted the number of pellets grasped with each hand. The hand with the highest score is considered as the preferred hand.

For the questionnaire, we calculated a handedness score by using the criteria of MacManus (2009):

*“Laterality scores (laterality indices)*:

Score all the items as −1 = Always left, −0.5 = Usually left, 0 = Either, +0.5 = Usually right and +1 = Always right. For items 4 (dish), 6 (jar), and 9 (potato) a strong right-hander would answer left. These three items should therefore be reverse scored by changing the sign on the values given previously (i.e., +1 = Always left, etc.). Having done this, then one can obtain the overall laterality score, an average of all 11 items.”

The score was then transformed into percentage (−100% indicating strongly left-handed and +100%, strongly right-handed).

The statistical analysis was conducted as follows. For the tube task, the reach and grasp drawer task, and the bimanual Brinkman board task, we used a binomial test (SPSS®; see Fig. 7). For the scores of the modified Brinkman board task, we used either the paired *t*-test or the Wilcoxon signed-rank test (Sigmastat®). Finally, for the CT derived from the modified Brinkman board task, we used either the unpaired *t*-test or the Mann–Whitney *U* test (Sigmastat®).

In order to limit the duration of the behavioral session with human subjects to a reasonable extent, the modified Brinkman board task using both hands simultaneously, as well as the reach and grasp drawer task, were not performed with human subjects. These tests, aimed in the monkeys to determine their preferred hand, were considered redundant for human subjects with the handedness questionnaire.

## Results

### Hand dominance: unimanual modified Brinkman board task

#### Monkeys

**Figure 2:**
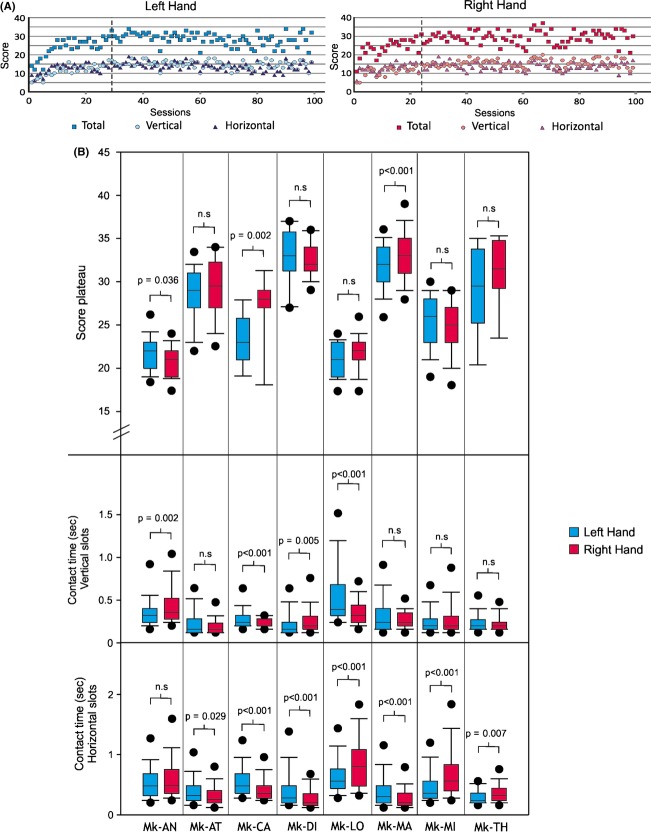
Hand dominance analysis for monkeys. An example of scores (Mk-AT) for the left and the right hand when the use of the hand was imposed in the modified Brinkman board task is shown in panel (A). Along the abscissa, the values refer to the consecutive daily session numbers, incremented by one for each individual session, irrespective of the actual date of the session. The regular interval between two consecutive sessions is thus not representative of the number of actual days separating the two sessions. In panel (B), three graphs in the form of box and whiskers plots represent for each monkey the distribution of the total scores (sum of horizontal and vertical slots) at the plateau (top graph), the distribution of contact times (CT, in seconds) for the vertical slots (middle graph) and for the horizontal slots (bottom graph), for the left hand (blue) and the right hand (red). These data concern the results when the use of one hand was imposed in the unimanual modified Brinkman board task. The statistical comparisons between the two hands in each daily session were performed using the paired *t*-test (normality test passed) or the nonparametric Wilcoxon rank signed test (normality test failed) for the score data (paired for the left hand and the right hand in a given daily session). In contrast, the CT data (five values per daily session for each slot orientation) are not paired and therefore the statistical comparisons between the two hands were performed using the unpaired *t*-test (normality test passed) or the nonparametric Mann–Whitney test (normality test failed) on the CT values pooled from 20 daily sessions.

For monkeys, the hand dominance was determined based on the total score in 30 sec (sum of vertical and horizontal slots in all behavioral sessions) and the CT (measured for the first five horizontal and the first five vertical slots) in the 20 last recorded sessions of the modified Brinkman board task, at plateau. The performance of one hand was compared to the performance of the other hand, measured in the two consecutive unimanual tests carried out on the same day. The dominant hand is the hand exhibiting a higher score, respectively, a shorter CT, than the opposite hand. For this specific analysis of hand dominance, only the score at plateau was taken into consideration (see Fig. 2A). A typical example of the score data is illustrated for one monkey (Mk-AT: left and right hand for total, vertical and horizontal slots) in Figure 2A, with a vertical dashed line separating the plateau phase from the preceding learning phase.

The top panel of Figure 2B represents the distribution of the scores for the left and the right hands for each monkey at plateau, in the form of box and whiskers plots. In Mk-DI, immediately after the end of the learning phase, there was a transient period with a decrease in the number of grasped pellets (most likely due to a temporary drop of motivation), corresponding to a first plateau. Later, the level of score corresponding to the end of the learning phase reappeared, corresponding to a second plateau, which was considered for the data of the top panel in Figure 2B. Overall, three monkeys exhibited a significant difference of manual dexterity reflected by the score between the hands, namely Mk-AN, Mk-CA, and Mk-MA. The first one performed better with the left hand (*P* = 0.036), whereas Mk-CA and Mk-MA were more dexterous with the right hand (*P* = 0.002 and *P* < 0.001, respectively). Mk-AT, Mk-DI, Mk-LO, Mk-MI, and Mk-TH did not show any significant difference of manual dexterity between hands at plateau, as far as the total score is concerned.

The CT data are plotted in the two bottom panels of Figure 2B. As the combination of movements required to grasp pellets were different for the two slot orientations, the CT was plotted separately for the vertical slots (middle panel in Fig. 2B) and for the horizontal slots (bottom panel in Fig. 2B). Overall, and as expected, the CTs for the vertical slots tended to be shorter (less challenging task) than the CTs for the horizontal slots. It is important to recall that the shorter the CTs, the better the performance. For the vertical slots, the CTs were significantly shorter for the left hand in Mk-AN and Mk-DI (*P* = 0.002 and *P* = 0.005, respectively), whereas they were significantly shorter for the right hand in Mk-CA and Mk-LO (*P* < 0.001 for both). For the other monkeys (Mk-AT, Mk-MA, Mk-MI, and Mk-TH), there was no significant difference of CTs between the two hands for the vertical slots. Considering the horizontal slots, the CTs were significantly different between the two hands for seven out of the eight monkeys, as only Mk-AN exhibited comparable CTs for the left and the right hand. In four monkeys (Mk-AT, Mk-CA, Mk-DI, and Mk-MA), the CTs were shorter for the right hand, whereas the CTs were shorter for the left hand in three monkeys (Mk-LO, Mk-MI, and Mk-TH). Considering both the vertical and the horizontal slots, note that in two monkeys (Mk-DI and Mk-LO) exhibiting a significant difference of CTs between the two hands for both slot orientations, surprisingly the hand with the shortest CTs was not the same for the vertical and the horizontal slots.

#### Human subjects

**Figure 3:**
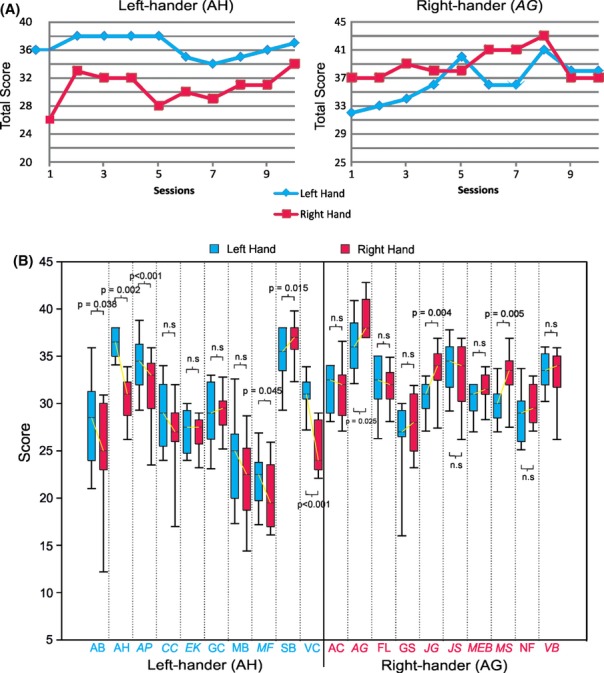
Hand dominance analysis for human subjects (women in italic), derived from the unimanual modified Brinkman board task. Examples of the total score (sum of the number of horizontal and vertical slots visited in 30 sec) for a left-handed subject (AH) and a right-handed subject (AG) are shown in panel (A). In panel (B), the box and whiskers plots represent the distribution of the total scores observed for the left hand (blue) and the right hand (red), for each human subject tested (*n* = 20, indicated by their ID initials). The ID initials of the subjects are in blue versus red, when the subjects presented themselves as left-hander versus right-hander, respectively. The ID initials of males and females are shown with normal and italic type, respectively. The statistical comparisons of total score between the two hands in each of the 10 trials were performed using the paired *t*-test (normality test passed) or the nonparametric Wilcoxon rank signed test (normality test failed). In each subject, a yellow line connects the median values of the left and the right hands, in order to emphasize the intermanual comparison.

The hand dominance was determined for the human subjects by comparing the total score (sum of vertical and horizontal slots visited in 30 sec) between each hand in the unimanual modified Brinkman board task. Graphs derived from one self-assessed right-hander (AG) and one self-assessed left-hander (AH) are shown in Figure 3A, with the total score for each hand in the ten consecutive trials. Generally, there was a training effect along the sessions, as most subjects increased their performance (total score) after a few trials. In two human subjects, the learning effect was rapid (plateau reached after two trials) but of limited extent (small increase of score). In the other human subjects, the learning phase was longer, 4–6 trials in most cases. The gain in total score was for most subjects in the order of 10 additional bolts collected in 30 sec at plateau as compared to the score observed for the first trial, although overall the gain in total score ranged from about 5–15 additional bolts collected in 30 sec. Moreover, most subjects developed strategies (motor habits) to increase their performance: for instance, they began to grasp bolts from the vertical slots and then bolts from the horizontal ones, or they began each trial on one side and systematically scanned the board to the other extremity. Additionally, in this sample of 20 human subjects, the right-handers performed significantly better than the left-handers (*P* < 0.001; Mann–Whitney test) and women exhibited higher total scores than men (*P* = 0.009; Mann–Whitney test).

The hand dominance was determined by comparing the total scores between the left hand and the right hand in each subject (Fig. 3B). Generally, the total score ranged between 15 and 40. Out of the twenty subjects, only nine showed a significant hand dominance. In the left-handed subjects (ID initials in blue in Fig. 3B; *n* = 10), five people exhibited a significant left-hand dominance: AB, AH, AP, MF, and VC (*P* = 0.038, *P* = 0.002, *P* < 0.001, *P* = 0.045, and *P* < 0.001, respectively), whereas one self-declared left-hander surprisingly showed a significant right-hand dominance (SB with *P* = 0.015). In the other four left-handers, there was no significant hand dominance. In the population of right-handed subjects (ID initials in red in Fig. 3B; *n* = 10), three of them showed a right-hand dominance (AG, JG, and MS, with *P* = 0.025, *P* = 0.004, and *P* = 0.005, respectively), whereas there was no significant hand dominance in the other seven self-declared right-handed subjects.

**Figure 4:**
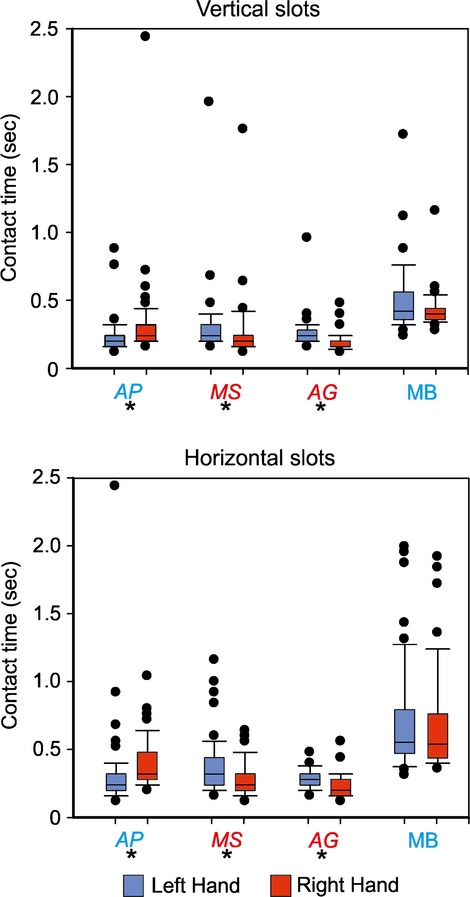
Hand dominance analysis for human subjects, derived from CTs obtained in the unimanual modified Brinkman board task, for four representative human subjects (see text), when the use of one hand was imposed. Both graphs, in the form of box and whiskers plots, represent the distribution of CTs in seconds, for the vertical slots (top graph) and for the horizontal slots (bottom graph), and separately for the left hand (blue) and the right hand (red). The CT data (five values per daily session for each slot orientation) are not paired and the statistical comparisons between the two hands were performed using the unpaired *t*-test (normality test passed) or the nonparametric Mann–Whitney test (normality test failed) on the CT values pooled from the 10 sessions. Same ID initial code as in Figure 3.

The CT was assessed in the human subjects as well, separately for the vertical and horizontal slots and illustrated in Figure 4 for four representative subjects. The subjects AP and MS were representative of lateralized humans, self-declared as left-hander and right-hander, respectively, and showed a dominance of the corresponding hand (left in AP and right in MS), with statistically shorter CTs as compared to the opposite hand. The CTs of two other subjects are displayed in Figure 4, one fast subject (AG) and one slow subject (MB), as exhibited in Figure 3B by their high and low scores, respectively. The fast subject (AG), declared as right-hander, also exhibited shorter CTs with the corresponding hand (the difference with the opposite hand was statistically significant only for the vertical slots). In contrast, the slow subject (MB), declared as left-hander, exhibited comparable CTs for both hands. As compared to monkeys (Fig. 2B), the human CT data (Fig. 4) reflect a somewhat shorter time interval needed to successfully grasp the object from the slots, especially for the horizontal slots. This species difference may be explained by the object properties, as the bolt with its angular contour and surface with a hole in it is easier to grasp than the round shape of the pellets presented to the monkeys.

### Hand preference

#### Monkeys

As reminder, the hand preference in monkeys was determined based on the results of the modified Brinkman board, when the use of the two hands was free, as well as on the results of three other specific tasks: the bimanual board, the tube, and the drawer tasks.

**Figure 5:**
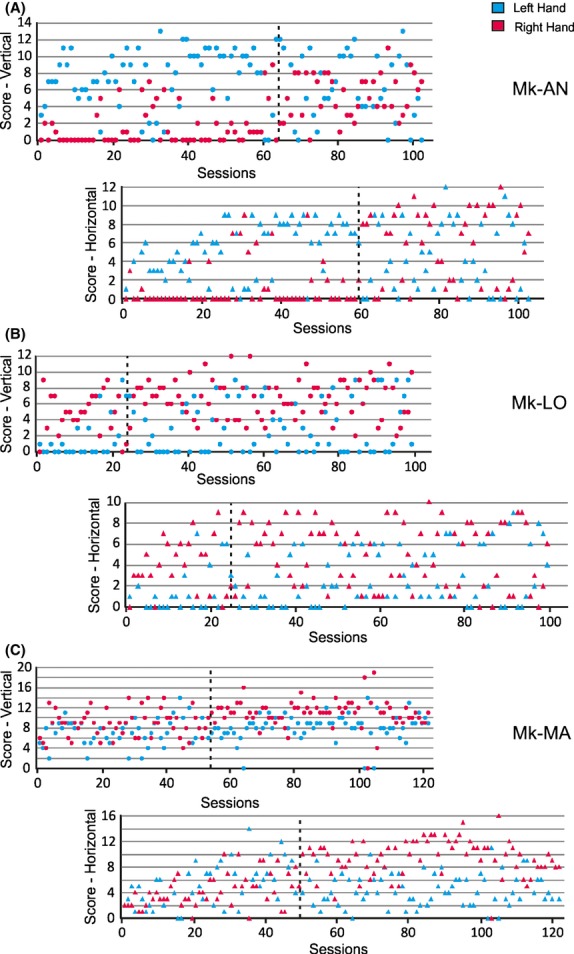
Hand preference in monkeys: distinction between different phases in the modified Brinkman board task, when the use of both hands was free. Different behaviors appear among monkeys. In panel (A), the scores for the vertical and horizontal slots for Mk-AN are shown. The vertical dotted line separates two phases: phase I in which the right hand (in red) was hardly ever used and phase II during which both hands were used more or less at the same frequency (see the corresponding statistical tests in Fig. 6). In panel (B), scores for vertical and horizontal slots for Mk-LO are shown. The vertical dotted line also separates two phases, but the distinction is here less marked. In phase I, the left hand was hardly ever used, whereas it was used more in phase II. However, the right hand seems to be more used in the two phases than the left one (see statistical tests in Fig. 6). In panel (C), scores for vertical and horizontal slots for Mk-MA are shown. The vertical dotted line separates two phases as well: phase I in which both hands were used more or less at the same frequency, and phase II, in which conversely the left hand was less used than the right hand (statistical tests in Fig. 6). As emphasis was put on the comparison between the two hands in each condition, the ordinate maximal values were variable among conditions.

For the modified Brinkman board task (executed with both hands simultaneously), we made a distinction among the scores according to different phases, each characterized by distinct patterns of manual use. Indeed, the monkeys evolved in their manner to execute the task and in the choice of one hand to the detriment of the other along the daily sessions. There were mainly three different behavioral profiles exhibited by the animals (Fig. 5). In the first profile (for instance Mk-AN in Fig. 5A), the monkey used nearly always the same hand in phase I, whereas in phase II (to the right of the vertical dashed line), both hands were used more or less at the same frequency. In the second profile (for instance Mk-LO in Fig. 5B), one of the hands was less used than the other hand along all daily sessions. However, two phases were distinguished, phase I corresponding to a minimal use of one hand followed, in phase II, by an increased contribution of the less used hand. The third profile (for instance Mk-MA, Fig. 5C) is the opposite to the first one: both hands were used more or less at the same frequency during phase I, whereas one hand was then less used than the other hand during phase II.

**Figure 6:**
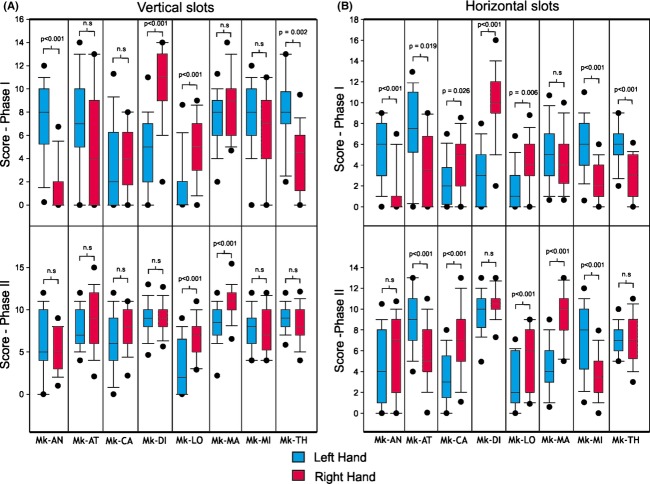
Hand preference statistical analysis for monkeys, applied to the modified Brinkman board task data, with free use of the two hands simultaneously, as illustrated in Figure 5, and represented by box and whiskers plots. Scores for vertical slots for phases I and II are shown for all monkeys in panel (A) and scores for the horizontal slots for phases I and II are displayed in panel (B).

After determining the different phases corresponding to different profiles (manual patterns), we compared the score for the right hand with the one for the left hand, separately in the vertical (Fig. 6A) and in the horizontal slots (Fig. 6B), in each phase in each monkey. In the vertical slots in phase I, four monkeys exhibited a significant preference to use one hand over the other (left-hand preference in Mk-AN and Mk-TH; right-hand preference in Mk-DI and Mk-LO), whereas the other four monkeys did not show any significant hand preference (Mk-AT, Mk-CA, Mk-MA, and Mk-MI). In phase II, most of the scores for the vertical slots did not exhibit a significant difference between both hands, except for Mk-LO and Mk-MA, with a significant preference for their right hand. In the horizontal slots (Fig. 6B), in phase I, all monkeys but Mk-MA showed a significant hand preference. Four monkeys (Mk-AN, Mk-AT, Mk-MI, and Mk-TH) used preferably their left hand, whereas three monkeys (Mk-CA, Mk-DI, and Mk-LO) used more often their right hand. In phase II, five out of eight monkeys showed a preference for one hand over the other, with a left-hand preference in Mk-AT and Mk-MI, whereas Mk-CA, Mk-LO, and Mk-MA exhibited a right-hand preference. Overall, there were clearly more significant hand preferences observed for the horizontal slots than for the vertical slots (Fig. 6).

**Figure 7:**
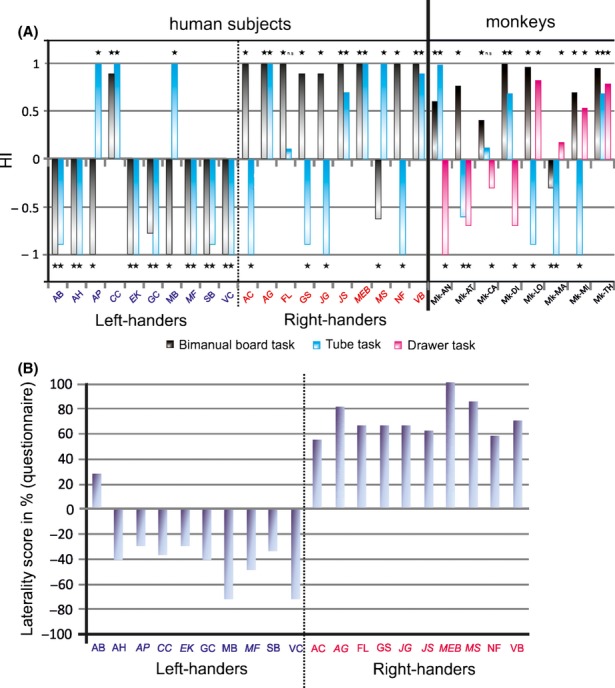
Hand preference analysis for monkeys and human subjects. In panel (A), the bar graph displays the handedness index (HI) for the bimanual Brinkman board and the tube tasks in human subjects and for the bimanual Brinkman board, the tube and the reach and grasp drawer tasks in monkeys. The solid vertical black line separates human subjects (left) from monkeys (right) and the vertical dotted line separates the human subjects who presented themselves as left-handers (left) from the subjects who presented themselves as right-handers (right). For each task and for each subject, the stars indicate a *P* ≤ 0.05 obtained in a binomial statistical test (ns = not significant, *P* > 0.05), above or below each corresponding bar graph. In panel (B), the bar graph represents the overall laterality score from the handedness questionnaire in percentage for each human subject. The ID initials of the subjects are in blue versus red for the self-announced left-handers versus right-handers, respectively. See text for statistical analysis. For human subjects, same ID initial code as in Figure 3 (women in italic).

The HI, derived from the three other tasks performed by the monkeys (the bimanual board task (Fig. 1B), the tube task (Fig. 1C), and the drawer task (Fig. 1D), were plotted on the same bar graph (Fig. 7A, rightmost part of the graph, separated from human subjects by a vertical black line). In most cases, these three tasks were lateralized (large positive or negative HI). Mk-TH was the only monkey to exhibit a coherent hand preference for all three tasks, with a systematically positive HI, corresponding to a significant right-hand preference (*P* < 0.05; binomial test). In the other seven animals, there was an absence of systematic consistency across tasks.

Three monkeys (Mk-AN, Mk-CA, and Mk-DI) exhibited a preference for the right hand in the bimanual board and the tube tasks (positive HI) and a preference for the left hand in the drawer task (negative HI). These HI values were statistically significant (meaning lateralized; binomial test *P* < 0.05), except in Mk-CA for the tube task (Fig. 7A).

Mk-LO and Mk-MI shared a comparable general pattern of HI distribution among the three tasks (Fig. 7A), namely a clearly positive HI (>0.5) for the bimanual board and the drawer tasks, whereas the HI was strongly negative for the tube task (Fig. 7A). In these two animals, all HI values were statistically significant (lateralized; *P* < 0.05).

The last three monkeys had each a unique general pattern of HI distribution among the three tasks. Mk-AT exhibited a significant preference for the right hand in the bimanual board task (*P* < 0.05), whereas a significant left-hand preference was present for the tube and the drawer tasks (*P* < 0.05). In Mk-MA, there was a significant left hand preference for the first two tasks (*P* < 0.05), whereas for the drawer task the right hand was preferred (*P* < 0.05).

#### Human subjects

Two tasks, namely the tube and the bimanual Brinkman board tasks, as well as the handedness questionnaire were used to assess the hand preference in human subjects. The observed HI values obtained for the bimanual board and for the tube tasks were plotted on the same graph for all subjects (Fig. 7A, left and middle parts of the graph, separated from the rightmost part concerning monkeys by the solid vertical black line). Most human subjects exhibited a HI near to −1 or 1. The *P*-value for each test and for each subject was statistically significant (*P* < 0.05; binomial test), except for the tube task in the subject FL (*P* > 0.05). The results for both tasks (Fig. 7A) showed that most self-declared left-handers indeed used their left hand as the preferred hand (HI negative), and similarly most self-declared right-handers indeed used their right hand as the preferred hand (HI positive). Only three left-handers exhibited a preference for the right hand in the tube task (subjects AP, CC, and MB). One of these three left-handed subjects (CC) furthermore showed a preference for the right hand in the bimanual board task. In the population of self-declared right-handers (Fig. 7A), four of them (subjects AC, GS, JG, and NF) showed a preference for their left hand in the tube task, whereas another right-handed subject (MS) exhibited a preference for the left hand in the bimanual board task. Statistical comparisons (*t*-test or Mann–Whitney) between the groups of right-handers versus left-handers for the tube task (blue bars in Fig. 7A) did not reveal any significant difference (*P* > 0.05) for both the real HI values and the absolute HI values. On the other hand, for the bimanual board task (gray bars in Fig. 7A), there was a significant difference for the real HI values between the right-handers and the left-handers (*P* = 0.002), but not for the absolute HI values (*P* = 0.33), indicating that the degree of lateralization is comparable in both groups.

The scores derived from the handedness questionnaire was calculated and transformed into percentages (Fig. 7B). The overall questionnaire scores for the self-announced right-handers (ID initials in red in Fig. 7B) were clearly positive, ranging between 53.85% and 100%. The questionnaire scores derived from the self-announced left-handers (ID initials in blue in Fig. 7B) were mostly negative, ranging between −30.77% and −73.08%. The exception was the subject AB, who surprisingly showed a positive questionnaire score (26.92%). The absolute values of laterality score were significantly larger in the right-handers than in the left-handers (*P* = 0.007), confirming the well-established notion that right-handers are more lateralized.

An overview of all results is available in Table 1, separately for the monkeys (Part A) and for the human subjects (Part B). Generally, it can be concluded that comparable numbers of left- and right-handed occurrences appeared among monkeys, concerning both the hand dominance and the hand preference (Table 1, Part A). However, there was no general consistency in hand dominance or in hand preference in monkeys, neither between individuals nor within each individual. On the contrary, as far as human subjects are concerned, the hand preferences revealed by the two manual tests and the questionnaire were largely coherent with the self-assessment by the subject (Table 1, Part B), although the tube task revealed a few more discrepancies. There were less systematic occurrences of hand dominance (assessed with the unimanual modified Brinkman board task; Table 1, Part B) although, when present, it was consistent with the lateralization of the hand preference (except in the subject SB). We also observed that hand dominance was somewhat more frequent in left-handers than in right-handers.

**Table 1:** Overview of the results. The panel (A) shows a summary of all results derived from the eight monkeys. VS and HS mean vertical and horizontal slots, respectively. Pl refers to plateau. Pl.I/Pl.II mean phases I and II of the plateau. The letter L indicates a left-hand dominance/preference and the letter R, a right-hand dominance/preference, whereas ns means a statistically nonsignificant difference (*P* > 0.05). The panel (B) shows an overview of all results in human subjects for the self-announced right-handers (ID initials in red) and left-handers (ID initials in blue). The letters L and R indicate a statistically significant left and right dominance/preference, respectively. ns means a statistically nonsignificant difference

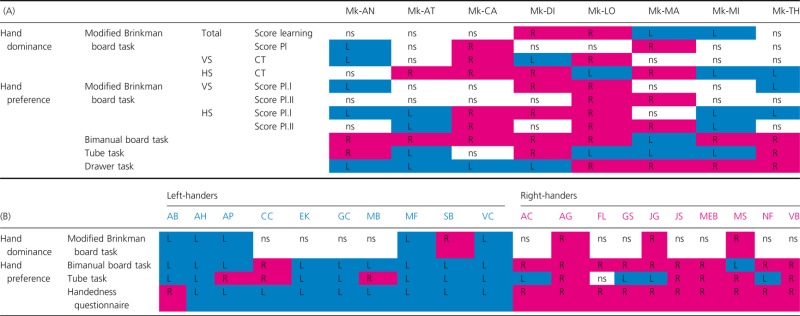

## Discussion

At least to the best of our knowledge, the present study introduced several new aspects of handedness assessment in primates, with emphasis on manual dexterity (use of precision grip). First of all, the data support the concept of separation of two hand attributes, namely the hand dominance and the hand preference. In monkeys, these two attributes were not systematically consistent, and in human subjects the hand preference was not systematically accompanied by consistent hand dominance, at least for the modified Brinkman board task (Table 1). This may be different for more challenging manual dexterity tasks. Second, the present study is original in comparing nonhuman primates and human subjects with respect to their handedness, based on a set of comparable manual dexterity tasks performed by macaque monkeys and human subjects (see also Lacreuse and Fragaszy 1997; for a comparison between capuchin monkeys and humans). In particular, the modified Brinkman board task widely and classically used in monkeys (e.g., Brinkman and Kuypers 1973; Brinkman 1984; Liu and Rouiller 1999; Kaeser et al. 2010, 2011, 2013; Schmidlin et al. 2011) was tested in human subjects for the first time. Third, the manual performance in nonhuman primates was conducted here in well-defined conditions, such as reproducible posture and position of the animal with respect to the behavioral set-up, thanks to the use of the primate chair placed in the same position from one daily session to the next (in contrast to observations in the wild or in the detention cage). The primate chair offers also the possibility to test separately the left hand from the right hand, as needed to assess hand dominance for instance. Finally, in monkeys, the assessment of manual performance was not restricted to a single or very few time points, but it was monitored in daily sessions over several weeks or months.

Overall, the results confirmed our hypothesis that hand preference in *M. fascicularis* is variable across manual tasks and individuals (Table 1). Furthermore, the hand preference in monkeys did not systematically correspond to the hand dominance in the modified Brinkman board task (four out of eight monkeys: see Table 1). In contrast, human subjects are more lateralized and the correspondence between hand preference and hand dominance was systematic in the vast majority of cases (one exception out of 20 subjects: see Table 1).

As expected, our results related to hand preference show that left-handers are not a mirror image of right-handers, at least based on the questionnaire (Fig. 7B). Right-handers are clearly more lateralized, as laterality scores (absolute values) were significantly larger in right-handers than in left-handers. In monkeys, based on the three tasks they performed (Fig. 7A), only one animal exhibited a consistent lateralization (Mk-TH: right-hander), whereas in the others, the preferred hand was largely task dependent.

The part of the present study focused on human subjects, in spite of a relatively limited sample of subjects (*n* = 20, comprising 10 men and 10 women distributed in 10 right-handers and 10 left-handers based on their self-assessment) revealed some interesting differences. First, the questionnaire data showed that left-handers are less lateralized than right-handers (Fig. 7B), as previously reported (see e.g., Kastner-Koller et al. 2007) and in line with our hypothesis (see Introduction and Methods). However, this lateralization difference between self-declared left- and right-handers reflected by the questionnaire was not found for the two bimanual tasks tested here: as shown in Table 1, there was a comparable number of hand preference deviations in each group (four right hand deviations in the left-handers and five left hand deviations in the right-handers). Second, in the context of hand dominance assessment based on the modified Brinkman board task, right-handers performed significantly better than left-handers, in the 10 trials conducted for each subject during the unique behavioral session. Whether this difference would be maintained along multiple sessions conducted at subsequent days remains an open question. Third, women performed significantly better than men in the modified Brinkman board task, as reflected by a higher total score. This result is in line with the previously reported notion that females perform better than males in tasks requiring high levels of manual dexterity (Kimura 2000). The gender difference was opposite in a computer-pointing task (Rohr 2006), with motor times shorter in men, favoring speed, than women, highlighting accuracy.

In the present study, fairly comparable results were obtained for human subjects and monkeys, as far as the hand dominance is concerned. Indeed, 62% of monkeys and 55% of human subjects did not show any statistically significant hand dominance, as assessed by the score derived from the modified Brinkman board task. Concerning the CTs, the results are more difficult to interpret in monkeys. The CTs were fully coherent with the score in one case only (Mk-CA), whereas for the other monkeys, there was no, or less, consistency (Table 1). As reminder, the CT is a parameter additional to the score, which eliminates possible biases in the score, due to inattention and/or lack of motivation of the monkey. In other words, it does not take into account the time interval between two slot manipulations. Moreover, we had taken into consideration only the last 20 sessions at plateau, to focus on the supposedly most stable daily behavioral sessions. It may, however, be interesting to consider the CT in more sessions in the plateau phase for a stricter comparison with the score for the very same sessions, although, in previous studies (e.g., Kaeser et al. 2010, 2011), the CTs were largely stable during the entire plateau phase. The discrepancy between score and CTs is likely to be due to other parameters, such as diverted attention in between the grasping of two consecutive pellets. It may also originate from the different motor habits reflected by the temporal sequence followed by the animal to visit the slots (e.g., the monkey scans the board systematically from one side to the other or from the middle and then to the sides; see Kaeser et al. 2013). Moreover, at a given time point, the animal may change prehension strategy (e.g., collect two pellets at a time). As long as the new strategy is not fully mastered, the hand dominance may vary, although the CTs remain short. In human subjects, as for the score data, the CT data showed that the hand dominance is generally consistent with the hand preference.

The present study offers the opportunity to compare the hand dominance and the hand preference for both human subjects and nonhuman primates. As reminder, the human subjects exhibiting hand dominance showed, most of the time, the same laterality for hand preference. This was not the case for the monkeys, where the laterality of the hand dominance did not systematically correspond to the one of the hand preference (Table 1). The same conclusion was met in a study conducted on four female *M. fuscata* Japanese monkeys (Kinoshita 1998).

Concerning the hand preference, the results in human subjects are very consistent with their self-assessment. Indeed, for most subjects, the preferred hand revealed by the different tasks corresponded to the hand they used to write, except for the tube task, where the results were more disparate (Table 1). The tube task thus appears less appropriate than the bimanual Brinkman board task and the questionnaire to determine the hand preference in human subjects. This raises then the question whether this task is adequate to assess hand preference in monkeys. The results related to hand preference in monkeys were highly disparate. Only two animals showed similar results (Mk-DI and Mk-AN) and, for each monkey, there was no systematic hand preference among all the tasks performed. Considering the questionable suitability of the tube task in human subjects (see above), it was tried to eliminate the tube test from the monkey data: omitting the tube task data did not modify substantially the results, except for Mk-LO, which was a right-hander for each task except the tube one. Two conclusions maybe drawn from these results: either the tasks used here are not fully appropriate to determine the hand preference in monkeys, or the *M. fascicularis* monkeys do not show a stable and systematic hand preference for the present panel of tasks. In human subjects, the bimanual Brinkman board appears to be an adequate test, but is it also the case for the nonhuman primates? This question highlights the limits of our experiment. On the one hand, we compare for the first time handedness in human subjects and in nonhuman primates for the same tasks directly but, on the other hand, these manual tasks may not be equally relevant in both species. The complexity and the representation of the different tasks may well be different for nonhuman primates and for human subjects. A difference is already present at the level of training. Clearly, human subjects reached more rapidly plateau values than monkeys, especially for the modified Brinkman board task. Human subjects are obviously more often engaged in bimanual coordination tasks in their everyday life than monkeys, a difference which may bias the comparison between the two groups performing the same manual tasks. At onset time of behavioral testing, the human subjects were already strongly lateralized, whereas this was most likely not the case in the nonhuman subjects. In the monkeys, the present data demonstrate that hand preference is more prominently revealed by a more challenging task (horizontal slots) than an easier task (vertical slots in the modified Brinkman board task, executed with both hands simultaneously; see Table 1). In the comparison between monkeys and humans, it has to be emphasized that reinforcement is not of the same nature (food in monkeys, a bolt in human) and therefore the motivational context is different. Furthermore, human subjects were asked to perform the task as rapidly as possible, whereas there was no such time constraint in monkeys. However, as the task represented the first access to food on that day, the monkeys were motivated and therefore they were fast too.

As compared to previous studies available in the literature, several aspects deserve further comments. As already mentioned above, few of the previous studies clearly distinguished hand dominance from hand preference, especially in nonhuman primates. Consequently, in previous studies conducted in monkeys with the aim to investigate the effect of different lesions of the central nervous system on the manual dexterity, it is often mentioned that a unilateral lesion was performed on the contralateral side with respect to the “dominant” hand. From the present study, such statement remains unclear as it is not obvious to distinguish whether the hand was more proficient (better motor performance reflecting hand dominance as defined here) or selected in priority (preferred hand) by the animal to perform a specific manual dexterity task. The difficulty is even increased when considering the data presented in Figure 5, demonstrating that the hand preference may vary with time along the daily behavioral sessions.

Focusing on hand preference (as defined in the present report), several studies showed similar results to ours, confirming an individual-level hand preference associated to different tasks (Old World Monkey in Westergaard et al. 2001a,b and Chapelain et al. 2006; Prosimians in Leliveld et al. 2008 and Hanbury et al. 2010). For Chapelain et al. (2006), this individual preference is an evidence of endogenous laterality, but to explain the differences between the animals, they propose an influence of different factors dependent on the task specificity. Hopkins (2006) reached similar conclusions in great apes. Linked to this observation, several studies suggested dependence between handedness and task complexity (Lehman 1989; Fagot and Vauclair 1991; Hopkins 1995; Hopkins and Rabinowitz 1997; Spinozzi et al. 1998; Hopkins and Cantalupo 2005). Indeed, the more complex the task, the more prominent the hand preference. This is in line with the larger occurrences of hand preference observed here in the horizontal slots of the modified Brinkman board task, as compared to the less challenging vertical slots (Table 1). Overall, in our study, all tasks in which the monkeys were engaged may be considered as complex, so it explains why, for most of them, we found an individual manual laterality (hand preference; see Table 1). Moreover, previous studies emphasized the significance of the body position in relation to the task in order to determine the manual laterality (Hopkins and Cantalupo 2005; Meunier et al. 2011). In our study, the position of the animal was highly reproducible and this parameter thus did not influence our results.

Unlike to the first aforementioned studies, Hopkins et al. (2002), Westergaard et al. (1997), and Wesley et al. (2001) found a population-level handedness in macaques and chimpanzees, but the methods used to assess hand preference were a bit different. Indeed, Hopkins et al. (2002) and Westergaard et al. (1997) tested the hand preference using a lower number of tasks.

Concerning the different results obtained from human subjects and monkeys, several explanations appear pertinent. Sociability plays an important role for the handedness (Hopkins 2006). Indeed, pedagogical or cultural pressures can influence the hand preference in humans, which is not considered to be the case in nonhuman primates. The postural origin theory of handedness offers a possible explanation for the monkey data (MacNeilage et al. 1987). Indeed, several studies showed a right-hand preference for more terrestrial species, whereas a left-hand preference was found for more arboreal animals (Masataka 1989; Singer and Schwibbe 1999; Hopkins et al. 2011; Meguerditchian et al. 2012; Zhao et al. 2012). In our case, our animal model, the *M. fascicularis*, is considered to be both arboreal and terrestrial (Fooden 2006; South Asian Primate C.A.M.P. Report, 2003; http://www.zooreach.org/downloads/ZOO_CAMP_PHVA_reports/2003%20Primate%20Report.pdf). Our results in *M. fascicularis* monkeys, showing a right- or left-hand preference depending on the tasks, is thus in line with the postural origin theory, in the sense that our animals did not show a clear right- or left-handedness, but an intermediate and variable position, consistent with the mixed arboreal and terrestrial status of *M. fascicularis*. These data are consistent with hand preference observations derived from simple food reaching task, also in cynomolgus (*M. fascicularis*) monkeys (Lehman 1980b). In a longitudinal study (from birth to weaning) conducted on a large number of monkeys (*M. fascicularis*), and based also on a task using a slot board but emphasizing more the attribute of hand dominance than hand preference (Brinkman and Smithson 2007), it was found that the infant monkeys showed a “dominant” hand at individual level (but bimodal distribution at population level). Their hand “dominance” was the same as that of their mother and, moreover, their pattern of grip movement resembled their mothers', suggesting imitation (Brinkman and Smithson 2007). In line with Hopkins (2004), the present data in *M. fascicularis* show that, as far as hand preference is concerned, they considerably diverge from human subjects (highly lateralized), whereas apes can be placed in between the two groups, with intermediate hand preference characteristics. This wide range of behavioral lateralization is consistent with its multifactorial origin (see e.g., Rogers 2009; Schaafsma et al. 2009; Uomini 2009; Forrester et al. 2013).
